# The prognostic value of preoperative serum levels of CA 19-9 and CEA in patients with pancreatic cancer.

**DOI:** 10.1038/bjc.1994.93

**Published:** 1994-03

**Authors:** J. Lundin, P. J. Roberts, P. Kuusela, C. Haglund

**Affiliations:** Fourth Department of Surgery, University of Helsinki, Finland.

## Abstract

The prognostic value of preoperative serum levels of CA 19-9 and CEA was evaluated in 160 patients with pancreatic cancer. The survival of patients whose tumour marker value was below a certain cut-off level was compared with the survival of those with a higher value using the log-rank test. The lowest cut-off level dividing patients into groups with significant difference in survival (P < 0.05) was determined by graphical analysis of chi-square values at different cut-off levels. If stage of disease was not taken into account, there was a significant difference in survival between patients with low vs high preoperative CA 19-9 and CEA levels. When patients were classified according to stage, a difference was found for CA 19-9 in stage II-III patients. Patients with preoperative CA 19-9 below 370 U ml-1 had a significantly better prognosis than those with a higher level (P < 0.05). In stage I and stage IV patients, no significant difference was found between the groups at any cut-off level. The analysis of CEA showed a significant difference in survival only in stage IV patients, with CEA above 15 ng ml-1 being associated with shorter survival. In conclusion, in patients with stage II-III disease, particularly in patients with a non-resectable tumour, in whom the exact spread of the disease may be difficult to evaluate even at operation, the preoperative CA 19-9 level seems to have a prognostic value.


Br. J. Cancer (1994), 69, 515–519 © Macmillan Press Ltd., 1994

### The prognostic value of preoperative serum levels of CA 19-9 and CEA in patients with pancreatic cancer

J. Lundin[1], P.J. Roberts[1], P. Kuusela[2] & C. Haglund[1]

[1]Fourth Department of Surgery and [2]Department of Bacteriology and Immunology, University of Helsinki, Helsinki, Finland.

**Summary** The prognostic value of preoperative serum levels of CA 19-9 and CEA was evaluated in 160 patients with pancreatic cancer. The survival of patients whose tumour marker value was below a certain cut-off level was compared with the survival of those with a higher value using the log-rank test. The lowest cut-off level dividing patients into groups with significant difference in survival ($P<0.05$) was determined by graphical analysis of chi-square values at different cut-off levels. If stage of disease was not taken into account, there was a significant difference in survival between patients with low *vs* high preoperative CA 19-9 and CEA levels. When patients were classified according to stage, a difference was found for CA 19-9 in stage II–III patients. Patients with preoperative CA 19-9 below 370 U $ml^{-1}$ had a significantly better prognosis than those with a higher level ($P<0.05$). In stage I and stage IV patients, no significant difference was found between the groups at any cut-off level. The analysis of CEA showed a significant difference in survival only in stage IV patients, with CEA above 15 ng $ml^{-1}$ being associated with shorter survival. In conclusion, in patients with stage II–III disease, particularly in patients with a non-resectable tumour, in whom the exact spread of the disease may be difficult to evaluate even at operation, the preoperative CA 19-9 level seems to have a prognostic value.

The overall prognosis of pancreatic cancer is poor, the 5-year survival rate being as low as 0.2–3.4% (Gudjonsson, 1987). Even after surgery for cure the 5-year survival in a meta-analysis was only 3.4% (Gudjonsson, 1987), although in a few recent studies higher survival rates have been reported (Trede *et al.*, 1990; Cameron *et al.*, 1991). The effect of chemotherapy in different studies has so far been limited. However, if patients are selected for adjuvant therapy, knowledge of factors influencing prognosis would be of great importance. Clinical stage is known to correlate with prognosis (Andren-Sandberg & Ihse, 1983). When comparing patients with the same stage of pancreatic cancer, very few prognostic factors are known. Histological type and grade correlate with prognosis, but in most pancreatic cancers only cytological specimens are available, and thus the differentiation grade is difficult to assess reliably.

It has been suggested that tumour markers might be used for evaluating prognosis in pancreatic cancer, but only a few studies have been reported. A positive correlation between low initial levels of CEA and CA 19-9 and survival has been described (Kalser *et al.*, 1978; Bottger *et al.*, 1990). However, in these studies stage of the disease was not taken into account.

The aim of this study was to evaluate the prognostic value of the preoperative serum levels of CA 19-9 and CEA in different stages of pancreatic cancer and to develop a method of determining the optimal cut-off level for prognostic evaluation.

### Patients and methods

### Patients

Serum samples were obtained from 160 patients with histologically or cytologically verified exocrine pancreatic carcinoma. Samples were taken preoperatively or at the time of diagnosis, and were stored at −20°C or −70°C until analysed. Stage of disease was based on data from clinical examination, imaging methods, operation records and surgical specimens. For stages I–III, post-operative mortality was excluded by excluding patients who died within 30 days from operation. One stage I patient was excluded, leaving 23 patients for analysis, and eight stage II–III patients were excluded and 49 analysed. All stage IV patients were evaluated, since the mean survival was extremely short and many patients did not undergo surgery at all. After exclusion of post-operative mortality, 151 patients were suitable for analysis. Preoperative CEA values were not available in 13 patients (five stage I patients, two stage II–III patients, six stage IV patients).

All stage I patients were operated for cure either by pancreaticoduodenal resection or total pancreatectomy.

Most of the patients with stage II–III disease underwent a palliative operation. In these operations local lymph nodes were not always removed adequately to allow differentiation between stage II and III. Of our patients, six clearly had stage II disease and 23 had verified lymph node metastases (stage III), whereas in 20 patients, local and regional lymph nodes were not removed for histological analysis. Therefore, patients with stage II and III disease were combined for analysis.

Seven stage II–III patients (six stage II, one stage III) underwent pancreaticoduodenal resection, 36 patients underwent palliative procedures, five patients underwent only diagnostic laparotomy and one patient was not operated on.

Thirty-five stage IV patients underwent palliative procedures, 12 patients diagnostic laparotomy, one patient diagnostic laparoscopy and 31 patients were not operated on.

Stages I–III were also analysed by dividing the patients into those with resectable and non-resectable disease.

Survival data of the patients were obtained from patient records, the Finnish Cancer Registry and the Population Registry.

### Assays

The serum concentrations of CA 19-9, defined by monoclonal antibody 1116 NS 19-9 against a human colonic carcinoma cell line (Koprowski *et al.*, 1979), and carcinoembryonic antigen (CEA), first detected in 1965 (Gold & Freedman, 1965) and today defined using monoclonal antibodies, were determined by commercially available CA 19-9 and CEA assays (Centocor, Malvern, USA; Abbott, Wiesbahn, Germany). The cut-off values recommended by the manufacturers for diagnostic purposes are 37 U $ml^{-1}$ for CA 19-9 and 3 ng $ml^{-1}$ for CEA.

Correspondence: C. Haglund, Fourth Department of Surgery, University Central Hospital, Kasarmikatu 11–13, SF-00130, Helsinki.
Received 6 July 1993; and in revised form 7 October 1993.

*Statistical analysis*

Analysis was performed using the Microsoft Excel program for Macintosh computers. Life tables were calculated according to Kaplan and Meier (1958). Patients were divided in groups having a preoperative tumour marker value above or below a certain cut-off level and their survival was compared. The statistical significance of the difference in survival of the groups was calculated using the log-rank test (Peto et al., 1977). Analyses were made for the whole patient material and separately for each stage group. Stage groups I–III were also analysed by dividing patients into those with resectable (23 stage I patients, six stage II patients and one stage III patient) and non-resectable disease (42 stage II–III patients). By gradually increasing the cut-off level, every achieved marker value was tested as cut-off point, searching for the lowest tumour marker level that would divide the patients in groups with a significant difference in survival. Graphs representing the log-rank chi-square values at the different cut-off points of CA 19-9 and CEA were created, and the cut-off values for prognostic evaluation were chosen as the lowest levels at which the chi-square value reached 3.84, corresponding to a significance level of $P<0.05$. This cut-off value was considered optimal for prognostic evaluation. Thus, patients with preoperative values above the cut-off level chosen will belong to a group with significantly worse prognosis compared with the patients with values below this level.

### Results

*Determination of the optimal tumour marker cut-off levels for evaluation of prognosis*

In stage II–III patients, a significant difference in survival between patients with marker value below vs above a certain cut-off level was reached at the preoperative CA 19-9 value of 370 U $ml^{-1}$ ($P<0.05$). When analysing only stage II–III patients with non-resectable disease the significance was even higher ($P<0.01$) (Figure 1). Since no significant difference in survival was seen for any other subgroup analysed, 370 U $ml^{-1}$ was chosen as cut-off level in all stage groups when evaluating CA 19-9 as a prognostic factor.

The analysis of CEA showed a significant difference in survival between patients with a preoperative value below 15 ng $ml^{-1}$ and those with a value above this level. In stages I–III, the differences in survival were not significant at any cut-off level. Therefore, 15 ng $ml^{-1}$ was chosen as cut-off level for prognostic evaluation of CEA in all stage groups.

*Survival according to stage*

In stage I, the median survival was 17.5 months, in stage II–III 8.6 months (16.3 months in resectable and 7.1 months in non-resectable disease) and in stage IV 2.3 months. The corresponding mean survival was 24.7 months, 10.9 months (17.9 vs 9.8 months) and 4.2 months respectively. The differences between the survival curves of the stage groups were highly significant ($P<0.001$), and the relative death rates calculated by the log-rank test were 0.42 for stage I, 0.81 for stage II–III and 1.96 for stage IV patients.

Also, dividing stage I–III patients into those with resectable and non-resectable disease and comparing these groups with each other and with stage IV patients, the differences in survival were highly significant ($P<0.001$) (Figure 2). The relative death rates calculated by the log-rank test were 0.42, 0.92 and 1.96 respectively.

*CA 19-9*

When analysing all patients, there was a significant difference in survival between those with a preoperative CA 19-9 level below (69 patients) 370 U $ml^{-1}$ and those whose level was above (82 patients) 370 U $ml^{-1}$ ($P<0.01$) (Table I).

**Figure 1** Log-rank chi-square values, corresponding to the significance of difference in survival between stage II–III (non-resectable) patients above vs below different cut-off levels of preoperative serum CA 19-9. The lowest cut-off level at which chi-square reached a value of 3.84 (dashed line), corresponding to $P<0.05$, was 370 U $ml^{-1}$.

**Figure 2** Life tables for patients with resectable (—) (23 stage I patients, six stage II patients and one stage III patient), non-resectable (--) (42 stage II–III patients) and advanced (---) (79 stage IV patients) pancreatic cancer.

**Table I** The median and mean survival times of patients with pancreatic cancer according to stage and preoperative serum CA 19-9 level

| | *Serum CA 19-9* <br> *<370 U ml⁻¹* | <br> *>370 U ml⁻¹* |
|---|---|---|
| Stage I | | |
| No. of patients | 16 | 7 |
| Median | 18.5 months | 17.5 months |
| Mean | 25.6 months | 22.8 months |
| Stage II–III resectable | | |
| No. of patients | 3 | 4 |
| Median | 16.6 months | 14 months |
| Mean | 22 months | 14.8 months |
| Stage II–III non-resectable | | |
| No. of patients | 21 | 21 |
| Median | 11.9 months | 5.2 months |
| Mean | 13.2 months | 6.5 months |
| Stage IV | | |
| No. of patients | 29 | 50 |
| Median | 2.7 months | 2 months |
| Mean | 4.8 months | 3.8 months |
| All patients | | |
| No. of patients | 69 | 82 |
| Median | 9.5 months | 4.4 months |
| Mean | 12.8 months | 6.7 months |

In stage I patients, the median survival was 18.5 months for patients having a CA 19-9 value lower than 370 U $ml^{-1}$ (16/23 patients), and 17.5 months for those with a higher value (7/23 patients). The corresponding mean values were 25.6 months and 22.8 months respectively. The difference between the survival curves was not statistically significant ($P > 0.05$) (Figure 3; Table I). Nor was there any significant difference when analysing all patients with resectable disease (stage I plus stage II–III resectable).

In stage II–III patients, the median survival for 24 patients with a level lower than 370 U $ml^{-1}$ was 13 months, compared with 6.4 months for 25 patients with a serum concentration greater than 370 U $ml^{-1}$. The difference between the survival curves was statistically significant ($P < 0.05$) (Table I).

When excluding stage II–III patients in whom the tumour was resected, and analysing only patients with non-resectable disease, the difference between survival curves of patients with a CA 19-9 level lower *vs* higher than 370 U $ml^{-1}$ was more significant ($P < 0.01$). The median survival for 21 patients with CA 19-9 below 370 U $ml^{-1}$ was 11.9 months compared with 5.2 months for 21 patients with a serum concentration greater than 370 U $ml^{-1}$. The corresponding mean values were 13.2 months and 6.5 months respectively (Figure 4; Table I).

In stage IV patients the median survival was 2.7 months for patients having a value lower than 370 U $ml^{-1}$ (29/79 patients) and 2 months for those with a higher value (50/79 patients). The corresponding mean values were 4.8 and 3.8 months respectively. The difference between the survival curves was not statistically significant (Figure 5; Table I).

### *CEA*

When analysing all patients, there was a significant difference in survival between those with a preoperative CEA level below (104 patients) *vs* above (34 patients) 15 ng $ml^{-1}$ ($P < 0.001$).

Only one stage I patient out of 18 had a preoperative CEA value above 15 ng $ml^{-1}$, and this did not allow statistically meaningful analysis. The same was true when analysing all patients with resectable disease.

Six out of 47 stage II–III patients had a preoperative CEA value above 15 ng $ml^{-1}$. All six patients belonged to the group of patients with non-resectable disease. The difference in survival between patients with CEA below *vs* above this value was not significant.

Of stage IV patients, 27 out of 73 had a CEA value higher than 15 ng $ml^{-1}$ and a median survival of 1 month compared with 3 months for those with a lower value. The corresponding mean values were 2 and 5 months. The difference was statistically significant ($P < 0.01$).

**Figure 3** Life tables for stage I patients with pancreatic cancer with preoperative CA 19-9 lower (—) or higher (---) than 370 U $ml^{-1}$. There was no significant difference between the groups.

**Figure 4** Life tables for stage II–III patients with non-resectable pancreatic cancer. Patients with a preoperative CA 19-9 level lower than 370 U $ml^{-1}$ (—) had significantly better prognosis than patients with CA 19-9 higher than 370 U $ml^{-1}$ (---).

**Figure 5** Life tables for stage IV patients with pancreatic cancer with preoperative CA 19-9 lower (–) or higher (---) than 370 U $ml^{-1}$. There was no significant difference in survival between the groups.

### *Combination of CA 19-9 and CEA*

When patients with preoperative CA 19-9 higher than 370 U $ml^{-1}$ and CEA higher than 15 ng $ml^{-1}$ were compared with patients who had marker values below these cut-off levels, the differences in the survival curves were not significant in any subgroup analysed. Nor were there significant differences in any group when comparing patients who had either CA 19-9 higher than 370 U $ml^{-1}$ or CEA higher than 15 ng $ml^{-1}$ with those who had lower marker levels.

### Discussion

In pancreatic cancer very few factors that influence prognosis are known. Stage of disease is known to correlate with survival, and this was also verified in our study. The differences in survival between the stage groups were highly significant. Also, dividing patients into clinically more relevant groups with resectable (stage I plus stage II–III resectable), non-resectable (stage II–III) and advanced disease (stage IV), the differences between the groups were significant.

Histological type and grade are also known to correlate with prognosis, but only in some patients is it possible to obtain adequate histological specimens even at surgery. Most pancreatic carcinomas are verified cytologically and the differentiation grade may be difficult to assess reliably from cytological specimens.

New prognostic markers would be of great value, and interest has recently been focused on immunological tumour markers. In this study, the prognostic value of CA 19-9 and CEA, two markers commonly used as diagnostic tests for pancreatic cancer, was evaluated.

Carcinoembryonic antigen (CEA), detected in 1965 by Gold and Freedman, was for more than a decade the only tumour marker of any clinical value in the diagnosis of pancreatic cancer. During the last 10 years it has been replaced by CA 19-9, which has a clearly higher sensitivity of 72–79% (Klapdor et al., 1984; Haglund et al., 1986; Steinberg et al., 1986; Benini et al., 1988). CA 19-9 is also sensitive in detecting recurrences of pancreatic cancer after operation for cure (Klapdor et al., 1984; Haglund et al., 1986, 1989). Both markers are also used as diagnostic tests for other forms of gastrointestinal cancer.

It has been suggested that preoperative levels of tumour markers could be used for evaluating prognosis, but very few studies have been published. For both CEA and CA 19-9 a positive correlation between a low marker level and survival has been shown (Kalser et al., 1978; Bottger et al., 1990). In neither study were the survival rates correlated with clinical stage, which is known to clearly correlate with prognosis. In this study, we evaluated whether high or low preoperative CA 19-9 and CEA levels might predict prognosis in patients of the same stage of pancreatic cancer.

We found it appropriate to exclude patients in stages I–III who died within 30 days of operation. Also, some other factors might alter the results, which is why different subanalyses were performed, as discussed below.

The CA 19-9 antigen corresponds to sialylated Lewis$^a$ blood group substance (Magnani et al., 1982). Therefore, it has been suggested that individuals lacking the Lewis gene (Lewis$^{a-\ b-}$), i.e. about 5–10% in different populations, are not able to produce the CA 19-9 antigen (Koprowski et al., 1982). Thus, the survival data of these patients might distort the overall results. On the other hand, Masson et al. (1990) found no difference in CA 19-9 expression between patients of different Lewis blood groups. Patients with a CA 19-9 level below the detection limit of the test ($<6.2\ U\ ml^{-1}$) might represent 'non-producers' of the antigen, although we did not determine the Lewis status of our patients. Excluding patients with CA 19-9 below $6.2\ U\ ml^{-1}$ from analysis did not alter the results of our study.

Elevated values of CA 19-9 and CEA are frequently seen in benign extrahepatic biliary obstruction, and it has been suggested that in patients with pancreatic cancer obstruction of the common bile duct might contribute to the elevation of serum marker levels (Barkin et al., 1978; Carr-Locke, 1980; Haglund et al., 1986, 1989; Barone et al., 1988; Paganuzzi et al., 1988). If the patients of this study had been further divided into patients with or without jaundice, the groups would have been too small for meaningful statistical analysis. However, no clear difference in survival was seen, when comparing patients presenting with jaundice (serum bilirubin $>20\ \mu mol^{-1}$) with those without jaundice.

For diagnostic purposes, the upper limits of normal, i.e. the recommended cut-off levels of different markers, were determined from sera of healthy individuals. This level was $37\ U\ ml^{-1}$ for the CA 19-9 assay used in this study and $3\ ng\ ml^{-1}$ for the CEA assay. When using these cut-off levels for prognostic evaluation, no significant differences in survival were seen within the different stage groups or between patients with a higher vs lower preoperative marker value. Since no established cut-off value for prognostic evaluation was available, we developed a computer program to determine the lowest cut-off value that divided the patients in two groups with a significant difference in survival. In stage II–III patients the optimal cut-off value was almost exactly $370\ U\ ml^{-1}$, which is tenfold greater than the cut-off level used for diagnostic purposes. Since no differences in survival between patient groups were found at any cut-off level in stages I and IV, the cut-off level of $370\ U\ ml^{-1}$ was chosen for further analysis.

Our study confirmed that there is a clear and significant difference in survival between patients with pancreatic cancer with a low vs high preoperative tumour marker level, if stage of disease is not taken into account. However, when patients were divided according to stage, the differences were significant only in stage II–III, both including and excluding patients with resectable disease, and only for CA 19-9. The difference in survival may reflect a difference in biological behaviour between tumours associated with a low level in contrast to a high level of CA 19-9. On the other hand, the stage II–III group is rather non-homogeneous, including patients with local disease, but with varying degree of spread to local lymph nodes. The size of the primary tumour may also vary markedly. Therefore, the difference in survival between patients with low and high CA 19-9 values might also reflect differences in tumour burden and spread of disease.

In stage I patients, the prognosis seemed to be independent of the preoperative serum expression of CA 19-9 and CEA, which was also true when analysing all patients with resectable disease. This may be explained by the fact that all patients underwent operation for cure, in which all macroscopic tumour tissue was removed. Therefore, postoperatively they may, from a prognostic point of view, be considered to be at the same starting point.

In stage IV patients, no difference in survival was seen between patients with a high or low CA 19-9 level, independently of the cut-off level chosen. This is probably explained by the very short overall survival of these patients. When stage IV patients were divided in two groups according to the preoperative CEA value, a difference was found in survival. However, this was true only for patients with extremely high preoperative values, above $15\ ng\ ml^{-1}$. In clinical practice, this finding is of limited value because of the very short survival in both groups, 5 months for patients with a value lower than $15\ ng\ ml^{-1}$ and 2 months for those with a higher value.

In conclusion, when evaluating prognosis, the preoperative serum level of CA 19-9 seems to be of some clinical value in stage II–III patients, in whom spread of disease may be difficult to assess even at surgery.

One explanation for the limited prognostic value of preoperative CA 19-9 and CEA levels might be the fact that the serum level of a tumour marker is not a reflection of the ability of the tumour tissue to synthesise the antigen, but rather a consequence of many other factors affecting the amount of circulating antigen. Among these factors are secretion of produced antigen into the bloodstream, metabolism and excretion of the antigen and occurrence of liver metastases. These factors are difficult to measure and the mechanisms are partly unknown. Immunohistochemistry seems a more reliable method of evaluating synthesis of the antigen, and the correlation between CA 19-9 staining and prognosis should be studied.

This study was supported by grants from Finska Läkaresällskapet, Medicinska Understödsföreningen Liv och Hälsa and the Karin and Einar Stroems Foundation.

### References

ANDREN-SANDBERG, A. & IHSE, I. (1983). Factors influencing survival after total pancreatectomy in patients with pancreatic cancer. *Ann. Surg.*, **198**, 605–610.

BARKIN, J., KALSER, M., KAPLAN, R., REDLHAMMER, D. & HEAL, A. (1978). Initial levels of CEA and their rate of change in pancreatic carcinoma following surgery, chemotherapy and radiation therapy. *Cancer*, **42**, 1472–1476.

BARONE, D., ONETTO, M., CONIO, M., PAGANUZZI, M., SACCOMANNO, S., ASTE, H. & PUGLIESE, V. (1988). CA 19-9 assay in patients with extrahepatic cholestatic jaundice. *Int. J. Biol. Markers*, **3**, 95–100.

BENINI, L., CAVALLINI, G., ZORDAN, D., RIZZOTTI, P., RIGO, L., BROCCO, G., PEROBELLI, L, ZANCHETTA, M., PEDERZOLI, P. & SCURO, L.A. (1988). A clinical evaluation of monoclonal (CA 19-9, CA50, CA12-5) and polyclonal (CEA, TPA) antibody-defined antigens for the diagnosis of pancreatic cancer. *Pancreas*, **3**, 61-66.

BOTTGER, T., ZECH, J., WEBER, W., SORGER, K. & JUNGINGER, T. (1990). Relevant factors in the prognosis of ductal pancreatic carcinoma. *Acta Chir. Scand.*, **156**, 781-788.

CAMERON, J.L., CRIST, D.W., SITZMANN, J.V., HRUBAN, R.H., BOITNOTT, J.K., SEIDLER, A.J. & COLEMAN, J. (1991). Factors influencing survival after pancreaticoduodenectomy for pancreatic cancer. *Am. J. Surg.*, **161**, 120-124.

CARR-LOCKE, D. (1980). Serum and pancreatic juice carcinoembryonic antigen in pancreatic and biliary disease. *Gut*, **21**, 656-661.

GOLD, P. & FREEDMAN, S. (1965). Demonstration of tumor-specific antigens in human colonic carcinomata by immunological tolerance and absorption techniques. *J. Exp. Med.*, **121**, 439-462.

GUDJONSSON, B. (1987). Cancer of the pancreas. 50 years of surgery. *Cancer*, **60**, 2284-2303.

HAGLUND, C., ROBERTS, P.J., KUUSELA, P., SCHEININ, T.M., MAKELA, O. & JALANKO, H. (1986). Evaluation of CA 19-9 as a serum tumour marker in pancreatic cancer. *Br. J. Cancer*, **53**, 197-202.

HAGLUND, C., KUUSELA, P. & ROBERTS, P.J. (1989). Tumour markers in pancreatic cancer. *Ann. Chir. Gynaecol.*, **78**, 41-53.

KALSER, M.H., BARKIN, J.S., REDLHAMMER, D. & HEAL, A. (1978). Circulating carcinoembryonic antigen in pancreatic carcinoma. *Cancer*, **42**, 1468-1471.

KAPLAN, E. & MEIER, P. (1958). Nonparametric estimation from incomplete observations. *Am. J. Statist. Assoc.*, **53**, 457-481.

KLAPDOR, R., KLAPDOR, U., BAHLO, M., DALLEK, M., KREMER, B., VAN, A.H., SCHREIBER, H.W. & GRETEN, H. (1984). CA 12-5 in cancer of the digestive tract. A comparison with CA 19-9 and CEA in cancer of the pancreas and colon. *Dtsch. Med. Wochenschr.*, **109**, 1949-1954.

KOPROWSKI, H., STEPLEWSKI, Z., MITCHELL, K., HERLYN, M., HERLYN, D. & FUHRER, P. (1979). Colorectal carcinoma antigens detected by hybridoma antibodies. *Somatic Cell Genet.*, **5**, 957-971.

KOPROWSKI, H., BROCKHAUS, M., BLASZCZYK, M., MAGNANI, J., STEPLEWSKI, Z. & GINSBURG, V. (1982). Lewis blood-type may affect the incidence of gastrointestinal cancer. *Lancet*, **i**, 1332-1333.

MAGNANI, J.L., NILSSON, B., BROCKHAUS, M., ZOPF, D., STEPLEWSKI, Z., KOPROWSKI, H. & GINSBURG, V. (1982). A monoclonal antibody-defined antigen associated with gastrointestinal cancer is a ganglioside containing sialylated lacto-N-fucopentaose II. *J. Biol. Chem.*, **257**, 14365-14369.

MASSON, P., PALSSON, B. & ANDREN, S.A. (1990). Cancer-associated tumour markers CA 19-9 and CA-50 in patients with pancreatic cancer with special reference to the Lewis blood cell status. *Br. J. Cancer*, **62**, 118-121.

PAGANUZZI, M., ONETTO, M., MARRONI, P., BARONE, D., CONIO, M., ASTE, H. & PUGLIESE, V. (1988). CA 19-9 and CA 50 in benign and malignant pancreatic and biliary diseases. *Cancer*, **61**, 2100-2108.

PETO, R., PIKE, M., ARMITAGE, P., BRESLOW, N., COX, D., HOWARD, S., MANTEL, N., MCPHERSON, K., PETO, J. & SMITH, P. (1977). Design and analysis of randomized clinical trials requiring prolonged observation of each patient. *Br. J. Cancer*, **35**, 1-39.

STEINBERG, W.M., GELFAND, R., ANDERSON, K.K., GLENN, J., KURTZMAN, S.H., SINDELAR, W.F. & TOSKES, P.P. (1986). Comparison of the sensitivity and specificity of the CA 19-9 and carcinoembryonic antigen assays in detecting cancer of the pancreas. *Gastroenterology*, **90**, 343-349.

TREDE, M., SCHWALL, G. & SAEGER, H.D. (1990). Survival after pancreatoduodenectomy. 118 consecutive resections without an operative mortality. *Ann. Surg.*, **211**, 447-458.


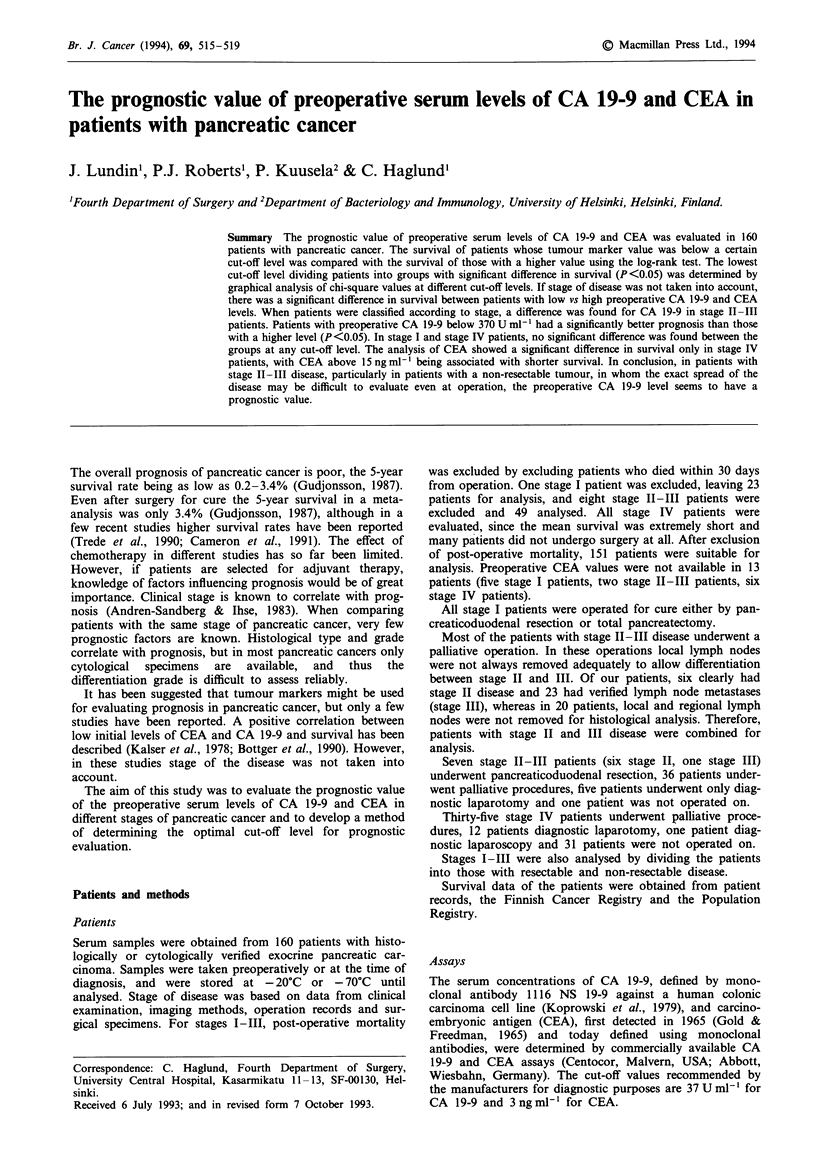

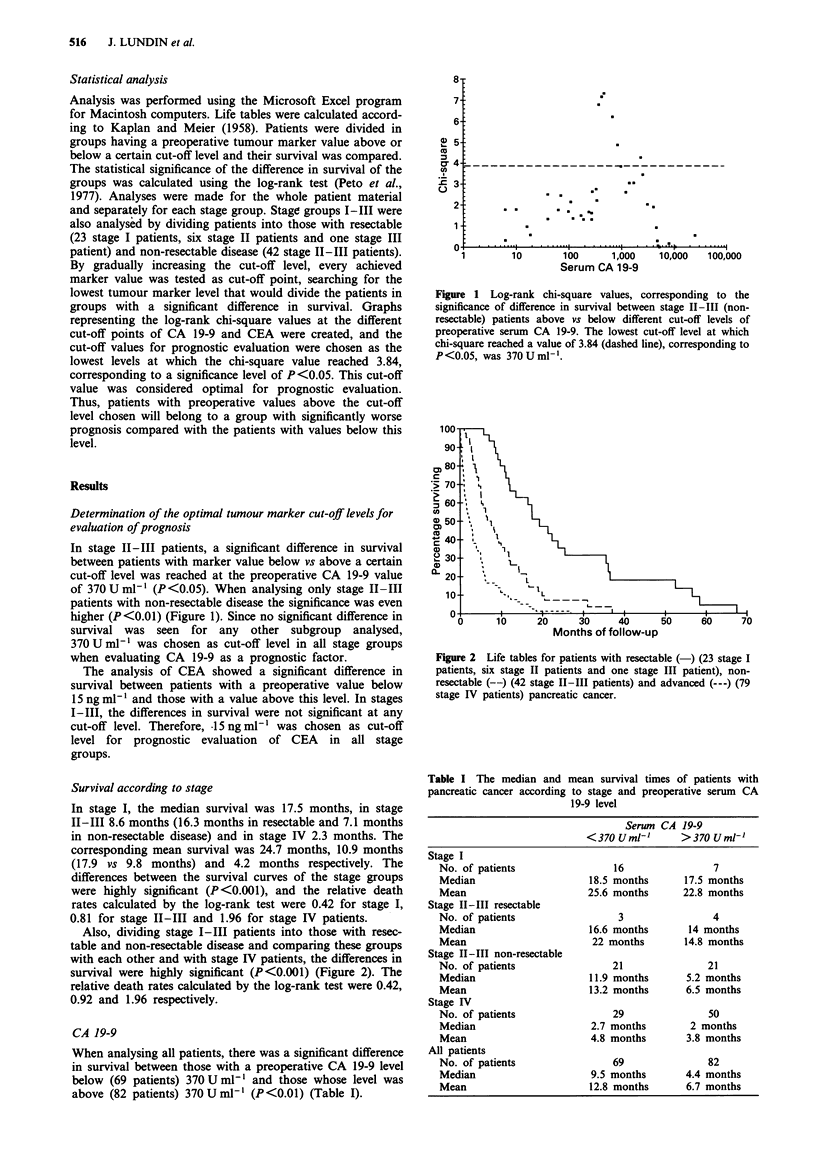

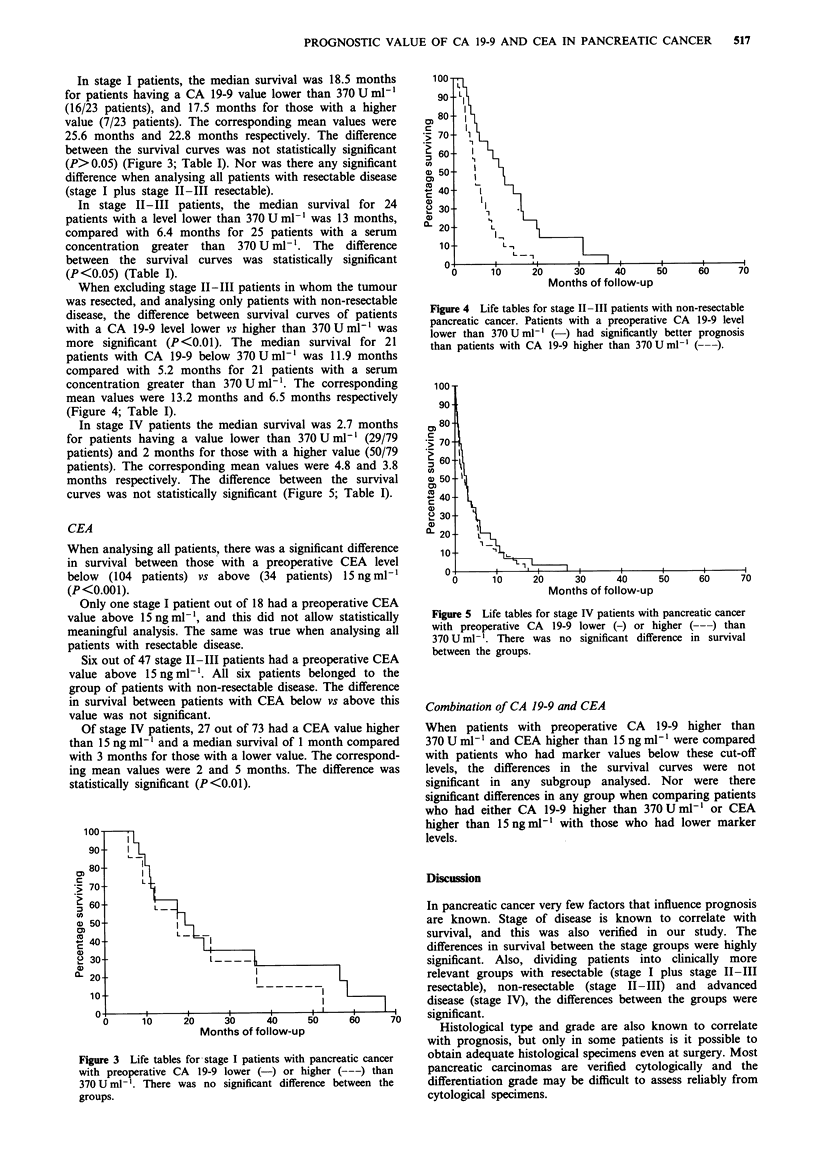

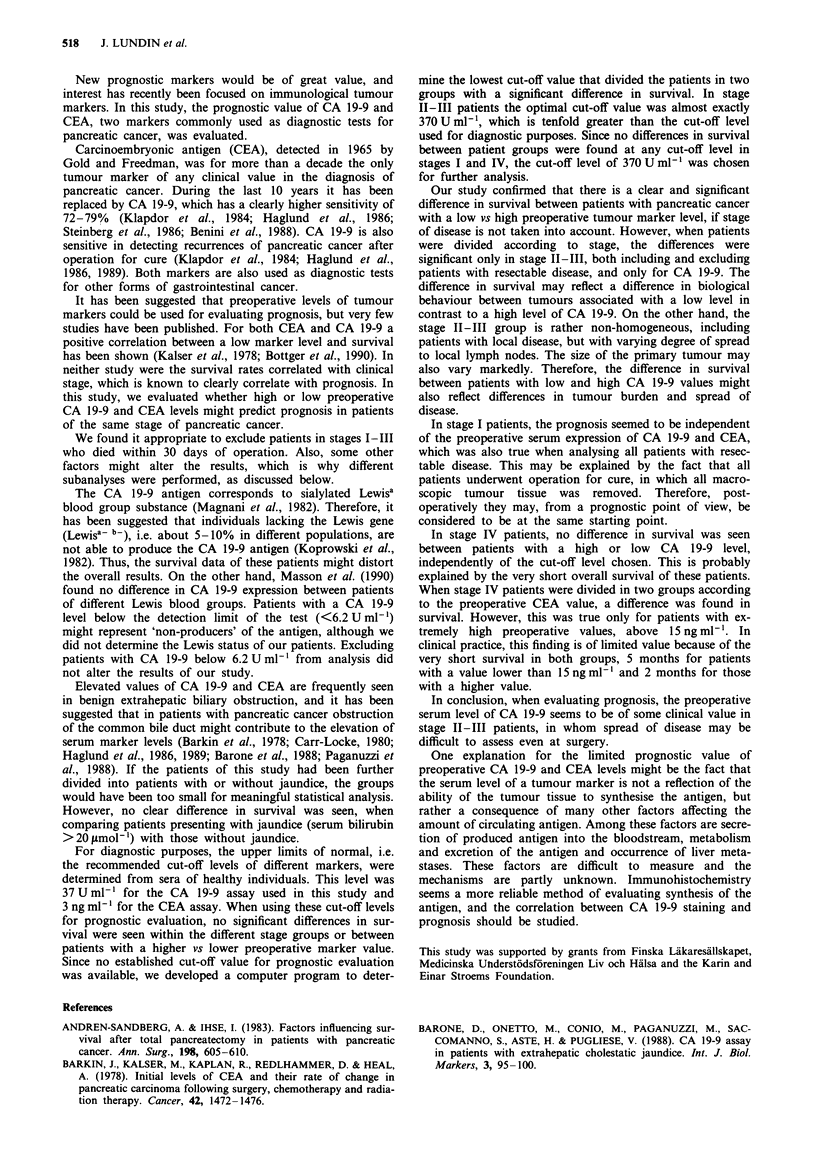

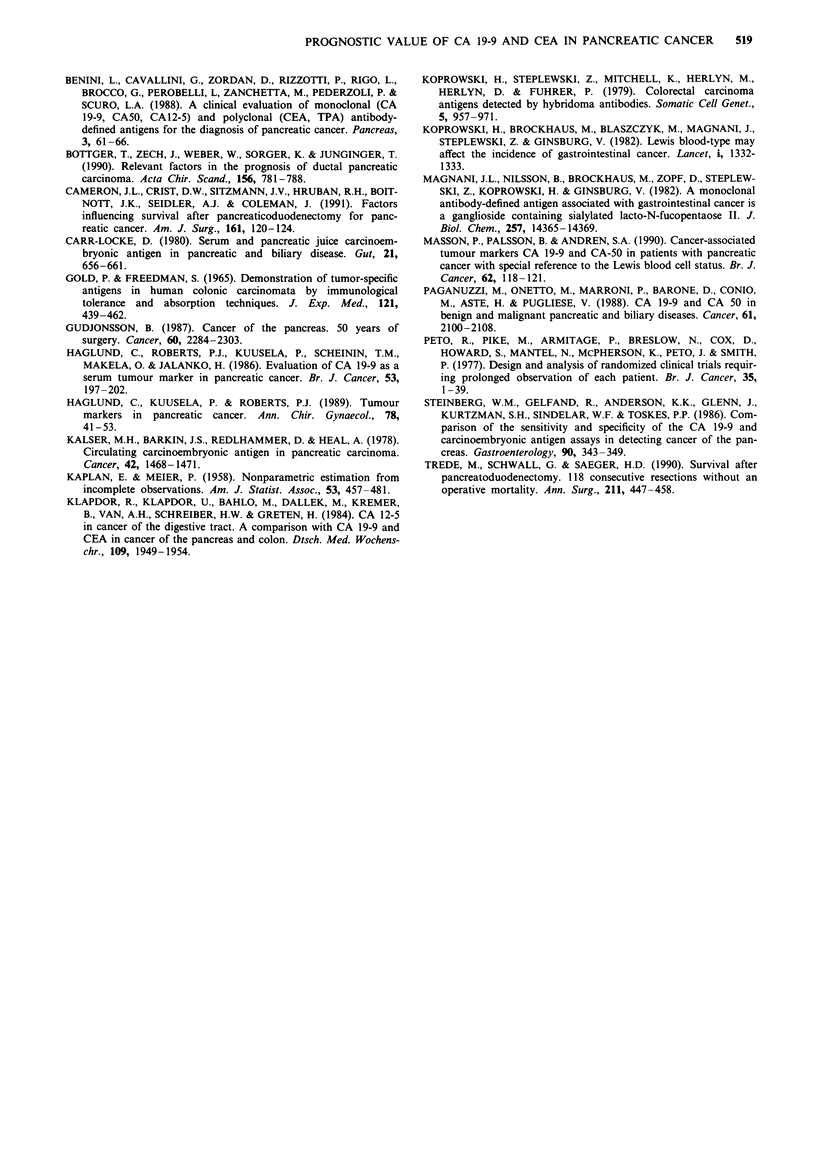


## References

[OCR_00669] Andrén-Sandberg A., Ihse I. (1983). Factors influencing survival after total pancreatectomy in patients with pancreatic cancer.. Ann Surg.

[OCR_00674] Barkin J. S., Kalser M. H., Kaplan R., Redlhammer D., Heal A. (1978). Initial levels of CEA and their rate of change in pancreatic carcinoma following surgery chemotherapy and radiation therapy.. Cancer.

[OCR_00682] Barone D., Onetto M., Conio M., Paganuzzi M., Saccomanno S., Aste H., Pugliese V. (1988). CA 19-9 assay in patients with extrahepatic cholestatic jaundice.. Int J Biol Markers.

[OCR_00688] Benini L., Cavallini G., Zordan D., Rizzotti P., Rigo L., Brocco G., Perobelli L., Zanchetta M., Pederzoli P., Scuro L. A. (1988). A clinical evaluation of monoclonal (CA19-9, CA50, CA12-5) and polyclonal (CEA, TPA) antibody-defined antigens for the diagnosis of pancreatic cancer.. Pancreas.

[OCR_00696] Böttger T., Zech J., Weber W., Sorger K., Junginger T. (1990). Relevant factors in the prognosis of ductal pancreatic carcinoma.. Acta Chir Scand.

[OCR_00703] Cameron J. L., Crist D. W., Sitzmann J. V., Hruban R. H., Boitnott J. K., Seidler A. J., Coleman J. (1991). Factors influencing survival after pancreaticoduodenectomy for pancreatic cancer.. Am J Surg.

[OCR_00707] Carr-Locke D. L. (1980). Serum and pancreatic juice carcinoembryonic antigen in pancreatic and biliary disease.. Gut.

[OCR_00712] GOLD P., FREEDMAN S. O. (1965). DEMONSTRATION OF TUMOR-SPECIFIC ANTIGENS IN HUMAN COLONIC CARCINOMATA BY IMMUNOLOGICAL TOLERANCE AND ABSORPTION TECHNIQUES.. J Exp Med.

[OCR_00718] Gudjonsson B. (1987). Cancer of the pancreas. 50 years of surgery.. Cancer.

[OCR_00728] Haglund C., Kuusela P., Roberts P. J. (1989). Tumour markers in pancreatic cancer.. Ann Chir Gynaecol.

[OCR_00722] Haglund C., Roberts P. J., Kuusela P., Scheinin T. M., Mäkelä O., Jalanko H. (1986). Evaluation of CA 19-9 as a serum tumour marker in pancreatic cancer.. Br J Cancer.

[OCR_00733] Kalser M. H., Barkin J. S., Redlhammer D., Heal A. (1978). Circulating carcinoembryonic antigen in pancreatic carcinoma.. Cancer.

[OCR_00742] Klapdor R., Klapdor U., Bahlo M., Dallek M., Kremer B., van Ackeren H., Schreiber H. W., Greten H. (1984). CA 12-5 bei Karzinomen des Verdauungstraktes. Ein Vergleich mit CA 19-9 und CEA bei Karzinomen des Pankreas und Kolon.. Dtsch Med Wochenschr.

[OCR_00755] Koprowski H., Brockhaus M., Blaszczyk M., Magnani J., Steplewski Z., Ginsburg V. (1982). Lewis blood-type may affect the incidence of gastrointestinal cancer.. Lancet.

[OCR_00749] Koprowski H., Steplewski Z., Mitchell K., Herlyn M., Herlyn D., Fuhrer P. (1979). Colorectal carcinoma antigens detected by hybridoma antibodies.. Somatic Cell Genet.

[OCR_00763] Magnani J. L., Nilsson B., Brockhaus M., Zopf D., Steplewski Z., Koprowski H., Ginsburg V. (1982). A monoclonal antibody-defined antigen associated with gastrointestinal cancer is a ganglioside containing sialylated lacto-N-fucopentaose II.. J Biol Chem.

[OCR_00768] Masson P., Pålsson B., Andrén-Sandberg A. (1990). Cancer-associated tumour markers CA 19-9 and CA-50 in patients with pancreatic cancer with special reference to the Lewis blood cell status.. Br J Cancer.

[OCR_00774] Paganuzzi M., Onetto M., Marroni P., Barone D., Conio M., Aste H., Pugliese V. (1988). CA 19-9 and CA 50 in benign and malignant pancreatic and biliary diseases.. Cancer.

[OCR_00780] Peto R., Pike M. C., Armitage P., Breslow N. E., Cox D. R., Howard S. V., Mantel N., McPherson K., Peto J., Smith P. G. (1977). Design and analysis of randomized clinical trials requiring prolonged observation of each patient. II. analysis and examples.. Br J Cancer.

[OCR_00787] Steinberg W. M., Gelfand R., Anderson K. K., Glenn J., Kurtzman S. H., Sindelar W. F., Toskes P. P. (1986). Comparison of the sensitivity and specificity of the CA19-9 and carcinoembryonic antigen assays in detecting cancer of the pancreas.. Gastroenterology.

[OCR_00794] Trede M., Schwall G., Saeger H. D. (1990). Survival after pancreatoduodenectomy. 118 consecutive resections without an operative mortality.. Ann Surg.

